# Fluoroscopy with MRA fusion image guidance in endovascular iliac artery interventions: study protocol for a randomized controlled trial (3DMR-Iliac-roadmapping study)

**DOI:** 10.1186/s13063-018-2981-0

**Published:** 2018-11-01

**Authors:** Seline R. Goudeketting, Stefan G. H. Heinen, Michiel W. de Haan, Anna M. Sailer, Daniel A. F. van den Heuvel, Marco J. van Strijen, Jean-Paul P. M. de Vries

**Affiliations:** 1 Department of Vascular Surgery, St. Antonius Hospital, Koekoekslaan 1, 3435 CM Nieuwegein, The Netherlands; 2 Department of Radiology, Maastricht University Medical Center, Maastricht, The Netherlands; 3 Department of Radiology, Stanford University School of Medicine, Stanford, CA USA; 4 Department of Radiology, St. Antonius Hospital, Nieuwegein, The Netherlands; 5 Department of Surgery, Division of Vascular Surgery, University Medical Center Groningen, Groningen, The Netherlands

**Keywords:** 3D imaging, Fusion imaging, Cone-beam computed tomography, Magnetic resonance angiography, Iliac artery, Angioplasty

## Abstract

**Background:**

Endovascular iliac artery interventions rely on the use of two-dimensional digital subtraction angiographies with an iodinated contrast agent and ionizing radiation. The amount of iodinated contrast agent should be limited because of its potentially nephrotoxic effects. Three-dimensional (3D) image fusion requires registration of a preprocedural magnetic resonance angiogram (MRA) or computed tomography (CT) angiogram to a perprocedurally acquired cone-beam CT or two fluoroscopic orthogonal projections. After registration, the 3D angiography images can be overlaid on the fluoroscopy screen and will follow table and C-arm movements. This study will assess the added value of the 3D image fusion technique in iliac artery interventions regarding the amount of the iodinated contrast agent administered.

**Methods/Design:**

The study cohort will comprise 106 patients (> 18 years) with symptomatic common and/or external iliac artery stenoses or occlusions and a recent (< 6 months) diagnostic MRA from the pelvis through the lower extremities, for which an endovascular intervention is indicated. Patients will be randomized into the control or study group (i.e. treatment without or with 3D image fusion guidance). The primary endpoint is the amount of administered iodinated contrast agent (mL). Secondary outcomes are technical success of the procedure, defined as < 30% residual stenosis over the treated lesion, fluoroscopy time, and radiation dose as dose area product (mGycm^2^). Patient participation in the study will be completed after hospital discharge.

**Discussion:**

This study is a randomized controlled multicenter trial to provide evidence on the effect of the 3D image fusion technique on the amount of administered iodinated contrast during endovascular common and/or external iliac artery interventions.

**Trial registration:**

Nederlands Trial Register, NTR5008. Registered on 16 December 2014.

**Electronic supplementary material:**

The online version of this article (10.1186/s13063-018-2981-0) contains supplementary material, which is available to authorized users.

## Background

The iliac arteries are involved in up to 30% of patients with peripheral artery disease (PAD) [1]. The preferred treatment for most patients is endovascular revascularization (i.e. percutaneous transluminal angioplasty [PTA] or recanalization) with optional stent placement. Two-dimensional (2D) digital subtraction angiography (DSA) with iodinated contrast agent and ionizing radiation is the gold standard to visualize the location and severity of the lesion [1]. Because of its potentially nephrotoxic effects, especially in patients with pre-existing renal failure or diabetes mellitus, the amount of iodinated contrast agent should be limited [2, 3].

**Fig. 1:**
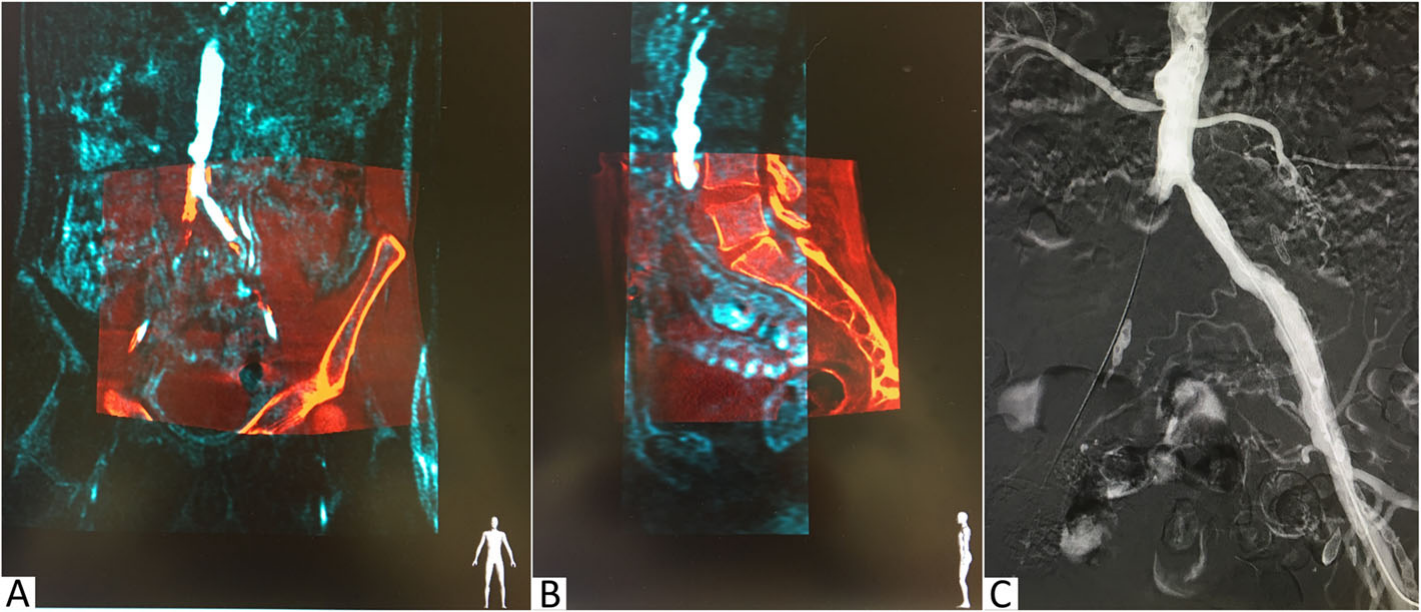
Example of fluoroscopy with MRA fusion image guidance. **a**, **b** Coronal and sagittal view of the image registration process (the cone-beam CT images in *red* and the MRA images in *blue*). **c** An example of the fusion image guidance during a digital subtraction angiography

Non-invasive imaging acquired before the procedure, such as contrast-enhanced magnetic resonance angiography (MRA) or computed tomography angiography (CTA), can be used as guidance during these endovascular interventions. The three-dimensional (3D) image fusion technique is based on the rigid registration of MRA or CTA to a perprocedurally acquired cone-beam computed tomography (CBCT) or, alternatively, two fluoroscopic orthogonal images. This registration is based on bony landmarks, after which the MRA images will be visualized on the 2D fluoroscopy screen (Fig. 1). Because of the registration, the MRA images will follow table and C-arm movements. The 3D image fusion technique does not require the use of additional iodinated contrast agent and has shown to significantly reduce the amount of iodinated contrast agent during endovascular abdominal aortic aneurysm repair (EVAR) [4–8].

No randomized controlled trials have been performed to assess the added value of the 3D image fusion technique in iliac artery interventions so far.

### Hypothesis

The hypothesis of this trial is that the use of the 3D image fusion technique will result in a significant reduction of the amount of administered iodinated contrast agent during endovascular iliac artery interventions compared with the endovascular iliac artery procedures without the use of the 3D image fusion technique.

## Methods/Design

### Study design

**Fig. 2:**
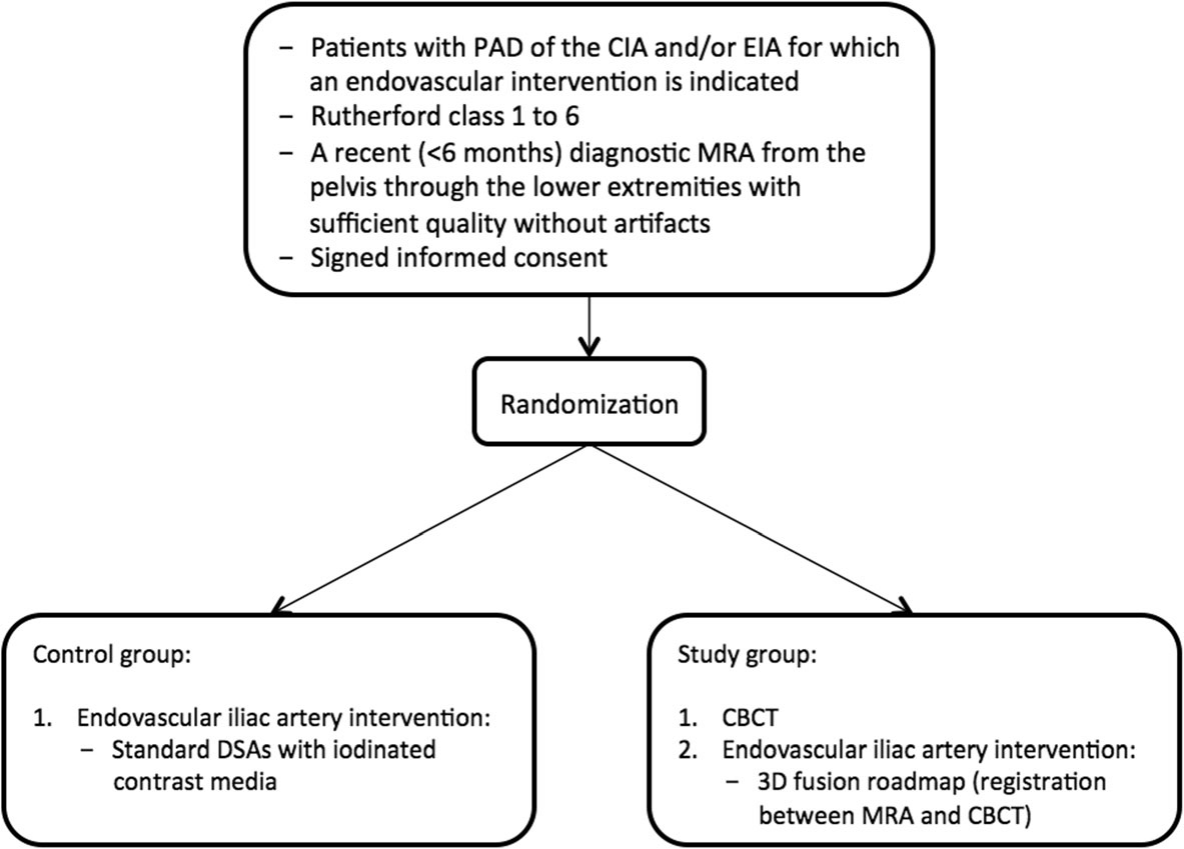
*Flow diagram* of the trial

**Fig. 3:**
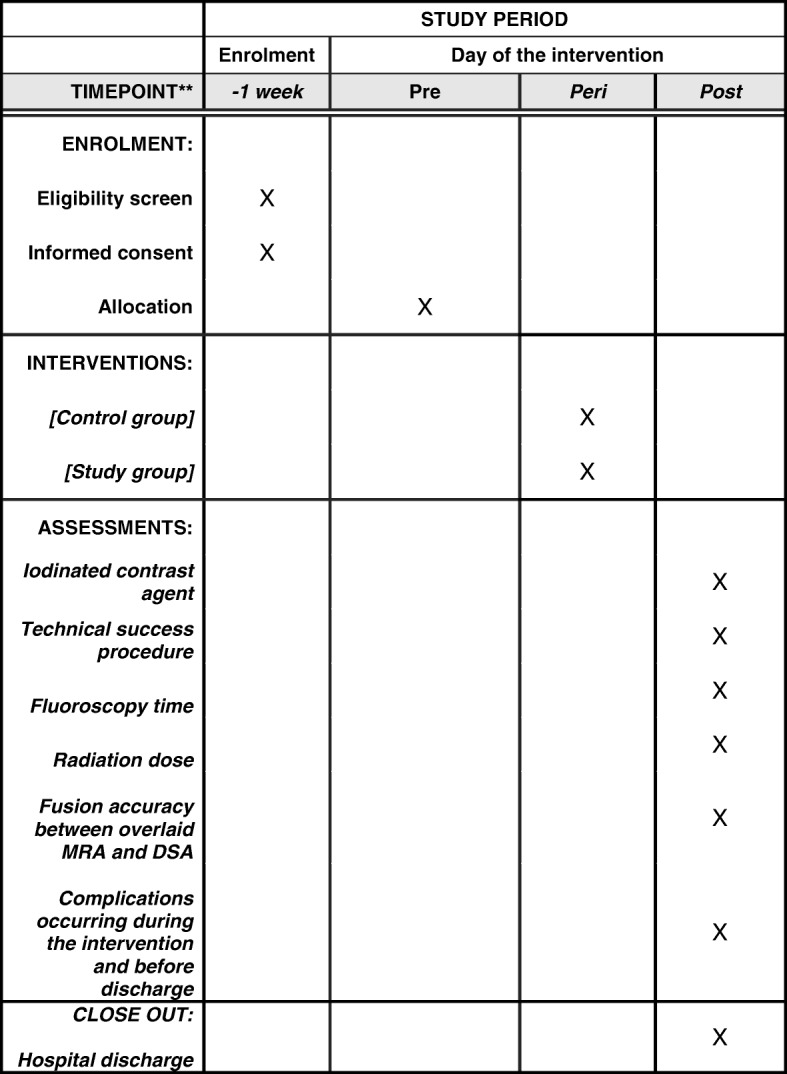
Schedule of enrolment, interventions, and assessments*

This is a prospective randomized, controlled, multicenter trial. The flow diagram of the trial is shown in Fig. 2. The SPIRIT figure can be found in Fig. 3 and the checklist is available in Additional file 1.

### Study objectives and endpoints

The primary endpoint is the amount of administered iodinated contrast agent (mL). The intra-arterial iodinated contrast agents Ultravist (300 mg/mL; Bayer Vital, Leverkusen, Germany) and Xenetix (300 mg/mL; Guerbet, Roissy, France) will be used during all interventions of the Maastricht University Medical Center (MUMC) and St. Antonius Hospital (SAH), respectively.

Secondary endpoints are the following:

1. Technical success of the procedure, defined as < 30% trans-lesion residual stenosis and no flow-limiting dissections;
2. Fluoroscopy time from the start to end of the procedure in minutes and seconds;
3. Overall procedure time, defined as the time from femoral access to arterial closure (minutes);
4. Radiation dose as dose area product (DAP) in mGycm^2^ from the start to the end of the procedure;
5. Fusion accuracy between the overlaid MRA and DSA, categorized as accurate (< 2 mm), mismatch (2–5 mm), or inaccurate (> 5 mm);
6. Complications that occur during the intervention and before discharge.

### Patients

The study cohort will comprise 106 patients (> 18 years) with symptomatic common iliac artery (CIA) and/or external iliac artery (EIA) stenoses or occlusions and Rutherford category 1–6, a recent (< 6 months) diagnostic MRA from the pelvis through the lower extremities, and an indication of endovascular intervention (PTA or recanalization). Participants will be randomized 1:1 to the control or study group (i.e. standard PTA or recanalization with or without the 3D image fusion technique). Patients will be included from the MUMC and SAH. Inclusion and exclusion criteria are summarized in Table 1.

**Table 1:** Eligibility criteria

Inclusion	Exclusion
Patients with PAD of the CIA and/or EIA for which an endovascular intervention is indicated	Age < 18 years
Rutherford class 1–6	Treatment for acute arterial ischemia
A recent (< 6 months) diagnostic MRA from the pelvis through the lower extremities with sufficient quality without artifacts (e.g. in stent restenosis or patient movement)	Allergy for iodinated contrast agents
Signed informed consent	Patients with aneurismal lesions of the iliac artery
	Pregnancy or breast-feeding

### Randomization

Randomization will be performed by the investigators with the use of an automated web-based randomization and electronic data capture software (MACRO 4.6.0, https://ecrf.ctcm.nl/macro). Randomization will be performed according to minimization; for a difference of 2 between the two groups (control and study group), there is a 90% chance that the next individual will be allocated to the group with the smallest number of patients. Stratification factors are the site (MUMC or SAH), weight (≤ 90 or > 90 kg), and estimated glomerular filtration rate (eGFR) (≤ 60 or > 60 mL/min/1.73m^2^). It is expected that the site, weight and eGFR have the biggest impact on the outcome measures. First, the groups should be similar regarding renal function because this may affect outcome. It can be argued that in patients with a reduced renal function, physicians are more careful with the amount of administered contrast. Thus, by using the eGFR as a stratification factor, this bias will be minimized. Second, the radiation dose is taken into account, which is dependent on the patients’ weight. Therefore, stratification on weight is important. Third, it is not preferable to have one of the sites primarily performing the interventions with the 3D fusion technique and vice versa, since that would influence the user experience and is possibly also be related to the amount of contrast volume that is administered and the radiation dose that is used. Hence, these stratification factors were chosen to keep the distribution between the groups equal. Owing to the nature of the study, randomization will not be blinded for patients or physicians.

### Ethics

The study will be conducted in accordance with the principles of the Declaration of Helsinki and the Medical Research Involving Human Subjects Act (in Dutch: Wet Medisch Wetenschappelijk Onderzoek met Mensen). The local medical ethical committees of both hospitals have approved the study protocol (NL47680.068.14, version 1.4). The study protocol was registered at the Netherlands Trial Register at www.trialregister.nl on 16 December 2014 (registration number: NTR5008). All patients will give written informed consent before study inclusion and randomization. Patient participation is voluntary; the participant can request to stop participation to the study at any time.

### Sample size calculation and statistical analysis

The sample size was calculated using a two-tailed t-test with null hypothesis *E(D) = 0* (no difference between the groups) and alternative hypothesis *E(D) ≠ 0* (groups are different). A type I error (*α*) of *0.05* with z-value *Z*_*α*_ ≈ *1.96* was accepted resulting in

1 $$ {Z}_{\alpha }=\sqrt{N}\frac{D}{\sigma_D}>1.96 $$

with *N* the sample size and *σ*_*D*_ the expected standard deviation. When assuming the alternative hypothesis is true *E(D) = τ* the power (*π*(*τ*)) can be calculated by

2 $$ \pi \left(\tau \right)=1-\Phi \left(1.96-\sqrt{N}\frac{\tau }{\sigma_D}\right) $$

with Φ being the Phi coefficient. When accepting a power of at least 0.90, the alternative hypothesis for *E(D) = τ* can be estimated by

3 $$ \pi \left(\tau \right)=1-\Phi \left(1.96-\sqrt{N}\frac{\tau }{\sigma_D}\right)>0.90. $$

Alternatively, this can be written with as

4 $$ \Phi \left(1.96-\sqrt{N}\frac{\tau }{\sigma_D}\right)<0.10. $$

For a type II error of 0.1, the z-value (*Z*_*β*_) becomes approximately *1.28*

5 $$ \sqrt{N}\frac{\tau }{\sigma_D}>1.96+{Z}_{\beta }=1.96+1.28\approx 3.24 $$

Given that half of the proportion of the study patients is randomized into the control group (*q*_0_ = 0.5) and the other half in the study group (*q*_1_ = 1 − *q*_0_ = 0.5), the sample size (*N*) can then be estimated by

6 $$ N>\left(\frac{1}{q_0}+\frac{1}{q_1}\right){\left(\frac{3.24}{\tau }{\sigma}_D\right)}^2 $$

Based on earlier research [9], we expect that *τ* = 20 mL of administered contrast volume may be reduced with the use of the 3D image fusion technique by reducing the number of contrast runs by at least two. Full-strength iodinated contrast is diluted 50:50 with a normal saline solution because a lower concentration of iodinated contrast agent has been shown not to compromise image quality during DSA of PAD patients [10]. To achieve a reduction of 20 mL per procedure with a mean volume of 60 mL and corresponding standard deviation of *σ*_*D*_ = 30 mL, the sample size *N* becomes a total of 96 patients. Therefore, 48 patients will be needed in each group. Adjusting for an anticipated loss to follow-up of 10%, the total sample size will be 106 patients. An interim analysis will be performed halfway through, after inclusion of the first 40 patients, to assess the efficacy of the 3D fusion technique compared to the control group.

Normal distribution of the data will be tested. Normally distributed data will be represented as the mean and 95% confidence interval; non-normally distributed data will be represented as the median and interquartile range. Continuous variables will be analyzed with the independent samples *t*-test or Mann–Whitney *U* test for normally and non-normally distributed data, respectively. Categorical data will be analyzed with the *χ*^2^ test. All tests will be two-sided and considered significant when *P* < 0.05.

### Intervention

The aim of the endovascular treatment is to obtain a patent CIA and/or EIA (i.e. < 30% residual stenosis or a reduction of the mean pressure gradient to < 10 mmHg [11]), resulting in an improved blood flow to the legs. In case of a flow limiting dissection, > 30% residual stenosis, or acute elastic recoil after PTA, additional stent placement will be performed. If indicated, other arteries will be treated in addition to the CIA and/or EIA during the same intervention. The physician will choose an ipsilateral or contralateral technique (or rendezvous procedure), depending on the location of the lesion. The analysis will not include data of patients with solely diagnostic DSAs.

Before the start of the intervention, the patient will be placed supine on the angiography table. The contrast pump and table contrast agent will be diluted 50:50 with a normal saline solution. For patients randomized into the study group, a non-contrast-enhanced CBCT will be created at the beginning of the intervention using the flat panel detector of the C-arm angiography system (Allura Xper FD20; Philips Healthcare). The CBCT will be automatically transferred to the 3D XtraVision workstation (Philips Healthcare). Subsequently, a manual 3D-3D registration will be performed between the preprocedural MRA and CBCT based on bony landmarks, such as vertebrae and the femoral heads, and the flow lumen on the MRA will be matched to the border of the calcifications as seen on the CBCT. The time required to perform the registration will be recorded.

After the registration, the physician will visually score the fusion accuracy of the entire iliac vasculature between the overlay and the initial DSA run. This fusion accuracy will be determined directly after the DSA is created and will be categorized as accurate (< 2 mm), mismatch (2–5 mm), or inaccurate (> 5 mm). An overlay that is inaccurate will be manually translated or reregistered to achieve a more accurate overlay that will be used during the intervention. Subsequently, the intervention will be performed according to the standard of care procedure and contrast runs will be performed when needed.

DSAs will be performed with 15 mL at 12 mL/s or 8 mL at 4 mL/s using a catheter in the aorta or CIA, respectively. A completion DSA will be created to confirm procedural success. DSA runs, whether or not in combination with the overlay (for the study group), will be stored. Primary and secondary endpoints will be registered, as well as baseline characteristics and procedural details, including ipsilateral or contralateral, length and location of the lesion, type of PTA balloon or stent, and number of stents.

All patients will receive 5000 IU of heparin during the intervention. The patients will receive bed rest after the intervention according to the physician’s prescription. All patients will be prescribed acetylsalicylic acid (ASA or aspirin) at 80 mg/d. The PTA and recanalization procedures are generally performed as outpatient procedures. Patient participation in the study will be completed after discharge. Any complication that occurs between the signed informed consent and hospital discharge will be recorded. There is no further follow-up period for this study.

### Data collection

Data will be collected by use of an electronic case report form (eCRF), which will be prospectively maintained from the time the patient signs the informed consent until the completion of the study. Patient data will be anonymized; each individual will be given a unique study number (site code [MUMC/ANTO] and index number). Each center can only view and add eCRF data from its own site. At the end of the study, the eCRFs of both sites will be become available to both centers.

### Monitoring and adverse events

A Data and Safety Monitoring Board (DSMB) will not be appointed for this study. A qualified monitor of the Clinical Trial Center Maastricht (CTCM) will monitor the study, which will ensure that the study is conducted according to ICH-GCP guidelines. Visits are conducted at the start of the study, after inclusion of five patients, halfway through, and at completion of the study. A monitoring report will be sent to the investigator after each visit.

Adverse events and serious adverse events will be recorded on the eCRFs. They will comprise a description of the event, type of complication (i.e. access site, systemic, organ-specific), severity, start date, end date, whether suspected to be intervention related, any action that is undertaken, and the outcome. Serious adverse events will be reported to the accredited METC through the “Toetsingsonline” page of the website of the Central Committee on Research Involving Human Subjects (in Dutch: Centrale Commissie Mensgebonden Onderzoek, CCMO [www.ccmo.nl]). The investigator will report serious adverse device events resulting from the use of the 3D image fusion technique to the accredited METC, the manufacturer, and the Inspection for Healthcare (in Dutch: Inspectie voor Gezondheidszorg [IGZ]).

## Discussion

The use of angiography during endovascular interventions is essential to visualize stenotic or obstructive lesions [12]. With increasing complexity of procedures, the amount of iodinated contrast agent may increase. The potentially nephrotoxic effects of the iodinated contrast agent are especially problematic in patients with pre-existing renal failure [2, 13, 14]. A recent systematic review confirmed that the amount of administered iodinated contrast agent can be significantly reduced by using the 3D image technique during EVAR procedures [15]. In addition, the radiation dose, procedure time, and fluoroscopy time may be reduced during these procedures [15], which could be beneficial to physicians, who receive high exposure to radiation throughout their practice.

A vacuum mattress can be used to reduce patient movement [16], which can minimize overlay inaccuracy caused by movement artifacts. We assume that movement artifacts will be smaller in the pelvic area than in the legs and that registration in this area will be less challenging because of the bony structures (vertebrae, femoral head, and iliac crest) and larger diameters of the arteries. This research focuses on the iliac arteries; when this study demonstrates the benefits of the 3D fusion technique in endovascular iliac artery interventions, the technique can be extended to femoro-crural lesions.

An alternative to iodinated contrast agents is the use of angiography based on carbon dioxide (CO_2_). The use of CO_2_ during EVAR procedures is safe and reliable and may be especially beneficial for patients with renal insufficiency [17]. However, variability in image quality has been reported and a careful technique is required for each injection to prevent air contamination. Additionally, there is a possibility of pain after injection, and CO_2_ for intravascular use has the potential for air embolism, which could result in ischemia or tissue infarction [18]. Although CO_2_ may be able to replace nephrotoxic contrast agents and reduce iodine contrast agent costs [18, 19], it will probably not reduce the radiation doses to patient and staff. Carbon dioxide and the 3D image fusion technique can be complementary during (complex) EVAR [20, 21]; however, 3D fusion in combination with CO_2_ for iliac artery interventions has not yet been investigated.

Preoperative MRA or CTA can be used for 3D image guidance. Registration between two similar image modalities (i.e. CTA to CBCT) may be less challenging than registration between MRA and CBCT, because MRA visualizes the flow lumen of the arteries, instead of calcified speculae. Nevertheless, because an MRA of the lower abdomen, pelvis, and lower extremities is the standard diagnostic imaging in the preoperative workflow of many patients with PAD, only patients with preoperative MRA datasets will be included; therefore, patients with contraindications to MRA are not eligible. Furthermore, because of susceptibility artifacts on the MRA, in-stent stenoses cannot be judged and those patients will not be included in this trial. No additional preoperative imaging needs to be performed for this study and patient burden is minimized.

Image registration can be performed to a CBCT (3D-3D image fusion) or two fluoroscopic orthogonal projections (2D-3D image fusion). The acquisition of a CBCT and registration process takes approximately 5–10 min and is reported to be more accurate than 2D-3D image fusion [20]. However, the latter is reported to be fast and can be performed with a minimal effective dose of 0.14–0.20 mSv [21]. Sailer et al. [22] reported that a CBCT corresponds to a mean effective dose of 3.5 mSv (95% confidence interval, 3.0–4.1 mSv), whereas van den Berg [21] reported an effective dose of 1.53–1.66 mSv. The patient’s radiation dose from a CBCT may add 21% of the total procedural DAP for iliac artery interventions [9], which is comparable to approximately 7 min of fluoroscopy time [22]. During fluoroscopy, the physician is at the tableside, but is usually far away from the C-arm during the acquisition of the CBCT. When the 3D fusion technique is used during EVAR, the fluoroscopy time could be significantly reduced [23, 24]. This reduction may also be feasible for iliac artery interventions, which will be beneficial to both the operator and the patient, and could potentially result in a reduction of costs. The possible benefit for contrast saving, combined with the possible reduction of fluoroscopy time might outweigh the additional radiation dose of a CBCT.

### Trial status

The METC has approved the study protocol. Currently, 40 patients have been enrolled in the 3DMR-Iliac-Roadmapping study. The expected project completion for this trial is December 2018.

## Additional file

Additional file 1: SPIRIT 2013 Checklist: Recommended items to address in a clinical trial protocol and related documents*. (DOC 121 kb)

## Abbreviations

2D: Two-dimensional
3D: Three-dimensional
ASA: Acetylsalicylic acid
CBCT: Cone-beam computed tomography
CCMO: Centrale Commissie Mensgebonden Onderzoek
CIA: Common iliac artery
CO_2_: Carbon dioxide
CTA: Computed tomography angiography
CTCM: Clinical Trial Center Maastricht
DAP: Dose area product
DCMB: Data and Safety Monitoring Board
DSA: Digital subtraction angiography
eCRF: Electronic case report form
EIA: External iliac artery
EVAR: Endovascular abdominal aortic aneurysm repair
IGZ: Inspectie voor Gezondheidszorg
MRA: Magnetic resonance angiography
MUMC: Maastricht University Medical Center
PAD: Peripheral artery disease
PTA: Percutaneous transluminal angiography
SAH: St. Antonius Hospital
